# Experimental Study of Diffusion Coefficients of Water through the Collagen: Apatite Porosity in Human Trabecular Bone Tissue

**DOI:** 10.1155/2014/796519

**Published:** 2014-05-21

**Authors:** Franco Marinozzi, Fabiano Bini, Alessandro Quintino, Massimo Corcione, Andrea Marinozzi

**Affiliations:** ^1^Mechanical and Thermal Measurements Laboratory, Department of Mechanical and Aerospace Engineering, “Sapienza” University of Rome, Via Eudossiana, 18-00184 Rome, Italy; ^2^Department of Astronautical, Electrical and Energetic Engineering, “Sapienza” University of Rome, Via Eudossiana, 18-00184 Rome, Italy; ^3^Orthopaedics and Traumatology Area, University “Campus Bio-Medico”, Via Álvaro del Portillo, 21-00128 Rome, Italy

## Abstract

We firstly measured the swelling of single trabeculae from human femur heads during water imbibition. Since the swelling is caused by water diffusing from external surfaces to the core of the sample, by measuring the sample swelling over time, we obtained direct information about the transport of fluids through the intimate constituents of bone, where the mineralization process takes place. We developed an apparatus to measure the free expansion of the tissue during the imbibition. In particular, we measured the swelling along three natural axes (length *L*, width *W*, and thickness *T*) of plate-like trabeculae. For this aim, we developed a 3D analytical model of the water uptake by the sample that was performed according to Fickian transport mechanism. The results were then utilized to predict the swelling over time along the three sample directions (*L*, *W*, *T*) and the apparent diffusion coefficients * D*_*T*_, * D*_*W*_, and * D*_*L*_.

## 1. Introduction

Transport phenomena within living tissues play an essential function for maintaining a proper supply of nutrients and for removing waste products. In bone tissue the transport of fluids and solutes is a concern for the bone formation and remodeling. These topics must be kept in mind when designing ECM-like scaffolds for tissue engineering to mimic the functions and structure of the biological materials [1].

Perhaps the most studied ECM with particular attention to its permeability and porosity is the bone tissue matrix. Bone is a dynamic and complex composite material with a composition of around 65 wt.% mineral phase, 25 wt.% organic, and 10 wt% water [2–8]. Referring to the volume fractions of the various parts, the bone volume (BV) is constituted by apatite minerals (33–43% BV) and organic constituents (32–44% BV) which are in turn composed of collagen type I (about 90%) and noncollagenous proteins (about 10%). The remnant is water (15–25% BV) [9] which plays a central role in the biomineralization process and contributes to the overall biomechanical properties of the biocomposite [10–12].

Water in bone may exist in three different forms: free water in pores, bound water in the collagen network (including collagen-mineral interface), and tightly bound water in the mineral phase [13, 14]. Bones with different water content display differences in stiffness and strength [15, 16]. Moreover, to describe the mechanical behavior of bone, the contribution of the bound water should be considered carefully by several viewpoints: first, the bound water in the collagen network will change the viscoelasticity of collagen phase dramatically [17, 18]; second, the bound water on the mineral-collagen interface will change the interfacial bonding properties [19]; third, water may migrate and change its local distribution within the collagen matrix in response to stress localization, consequently introducing the nonuniform properties into the collagen phase.

Transport phenomena through the bone tissue are dictated by its hierarchical structure with four levels of porosity: collagen-apatite (~10 nm), lacunar-canalicular (~100 nm), vascular (~50 *μ*m), and the intertrabecular porosity (~1 mm) [20]. The dimensions of the smallest pores (collagen-apatite porosity) are imposed by the intermolecular collagen bonds, that is, cross-links, the water content, and the degree of mineralization [21–24].

The water in the collagen structure was classified into 5 regimes [25] characterized by increasing water concentration from 0–0.010 g/g (regime I) to >0.5 g/g (regime V).

For the purposes of the present study it is important to point out that rehydrating the specimens from regime III causes a consistent large increase of the lateral spacing of the collagen molecules and thus produces a measurable swelling [26].

A small amount of studies was reported to our knowledge about the water dynamics and subsequent hygroexpansion in bone matrix. Diffusion coefficient [27] and water distribution [28] were measured by NMR, respectively, on rabbit and human cortical bone, while for trabecular bone few data about dimensional changes [29] and diffusion coefficient of single human trabeculae [30–33] are available.

The main scope of this work is to give experimental evidence of the fluid transport dynamics through the collagen-apatite porosity. To do that, the hygroexpansion of single human trabeculae consequent to water sorption was measured by means of a specially designed high accuracy dilatometer, and the results have been processed with the aid of a genetic algorithm.

The authors believe that this experimental investigation of transport phenomena within the bone matrix can offer a valuable support to the design of ECM-like scaffolds for bone tissue engineering. In fact, it is not sufficient for the scaffold to have the correct pore size but it should also exhibit proper connectivity to assure metabolic exchanges within the regenerating tissue.

## 2. Materials and Methods

### 2.1. Specimens Preparation

Four bone specimens of cancellous bone were extracted from human femur head withdrawn from a donor (female: age 67) suffering from moderate coxo-arthritis (CA). Its caput was substituted by hip arthroplasty surgery. From preliminary dual-energy X-ray absorptiometry (DXA) a slight degree of osteopaenia was found. The bone tissue volume measured by a micro-CT apparatus [34] confirms substantially this finding. We did not use cadaveric femoral heads because it seemed important to evaluate the specimen without possible postmortem changes. After identification, the bone specimens were stored at −10°C for one month then, from the frontal-plane middle-site of the femoral head, a 10 mm thick slice was obtained for each bone specimen and stored again at −10°C for 10 hours. Subsequently, the slices were defatted by means of three complete cycles of dehydration with aqueous solutions with an increasing percentage of ethanol, 70%, 90%, and 99.9%, respectively. Between the dehydration cycles specimens were stored at −10°C for 10 hours. Bone specimen slices were then cut with a diamond saw (EXTEC Labcut 1010, Enfield CT) in order to obtain 10 × 10 × 10 mm blocks, which were further dehydrated and defatted by other three cycles with ethanol solutions at different concentrations, as previously described. Each single trabecula was dissected from a block corresponding with one of the main trabecular groups, coincident with the principal stress trajectories in the loaded femur, according to the well-known Wolff's law [35]. A dissecting microscope was used to locate uniform trabeculae which were excised with a scalpel. In order to avoid this measurement error, great care was adopted in excising a single trabecula; that is, the specimen was taken by extracting the portion located between two adjacent struts. The dimensions of the dissected trabeculae were measured with a vernier caliper (resolution 0.05 mm) prior to each test.

A total of 23 single trabeculae were initially prepared. Eight specimens presented general defects or broke up in the attempt to pin them to the test machine. Six specimens were used to gain experience with the test machine in order to obtain a preliminary estimate of the swelling behavior. Five specimens showed anomalous swelling, probably due to some microcracks created during the preparation and/or dissecting procedure, and were discarded. Thus only 4 single trabeculae were tested for the trials.

### 2.2. Experimental Set-Up

**Figure 1:**
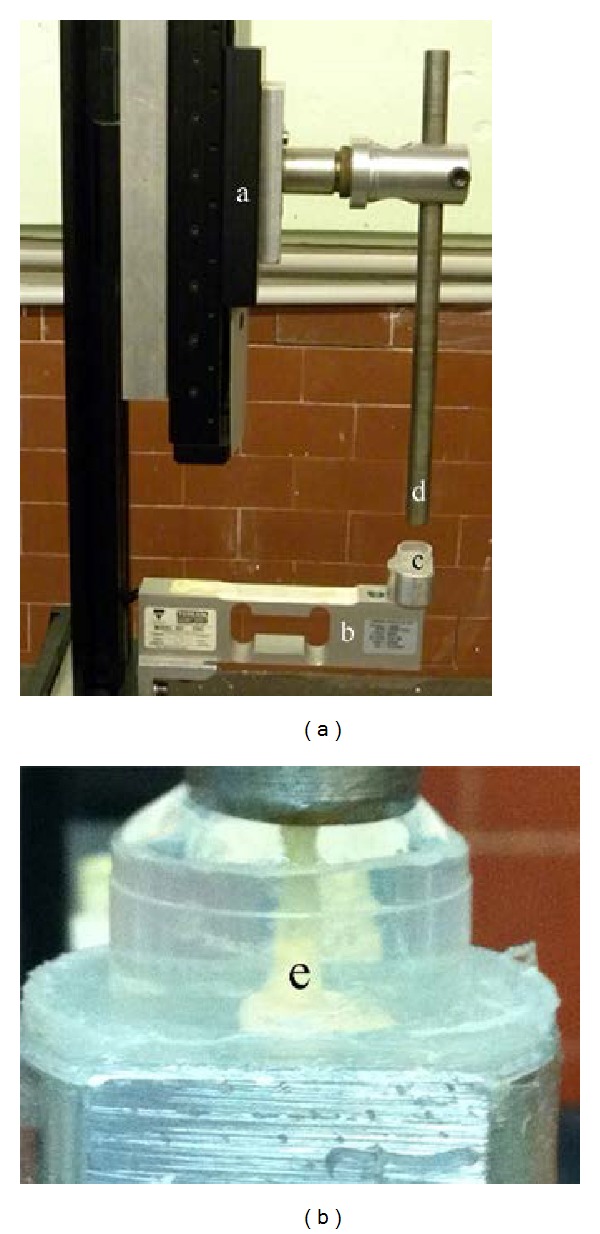
The apparatus for the measurement of swelling of a single trabecula (e). Microtranslation stage (a); load cell (b); distilled water filled container (c); and pushing rod (d) for the imbibition of the specimen.

Referring to Figure 1, to measure the free expansion of the specimen during the imbibition, we developed* ad hoc* apparatus (APP) composed of a micropositioning stage (a) (M-410 DG, ±0.2 *μ*m repeatability, Physik Instrumente, Germany) equipped by a steel rod and a strain gage load cell (b) (50 N full scale Vishay M1042-HBM, Germany). The APP was mounted in vertical position in order to accommodate a cylindrical container (c) of about 10 mL onto the free end of the load cell. Data sensed by the load cell was introduced into the strain gage amplifier (KWS 501A HBM, Germany), whereas the micropositioning is connected via RS232 serial cable to C-863 DC motor controller (Physik Instrumente, Germany).

During the start-up the steel rod was lowered on top surface of the specimen with a preload of 0.1 ± 0.01 N (mean value ± standard deviation). This preload was applied to assure proper but gentle fixation on top surface of the specimen between the load cell and the tip (d) of the pushing rod. Subsequently, the specimen was rapidly and totally submerged in distilled water.

As soon as the specimen begins to swell the micropositioning was moved upward by a feedback control system, processed by PID controller based on algorithm in NI Labview (National Instruments, Texas, USA) which actively maintains the applied preload at its constant value. The user sets a preload value due to actual demand, that is, the setpoint; as a consequence the current load cell signal acquired can be controlled to approach the set preload point. The output value of feedback loop controls the upward displacement of the micropositioning which measures the elongation in the axial direction of the trabecula caused by the hygroexpansion versus time.

A total number of 12 swelling trials were performed on the four specimens along three axial directions (*L*,* W*, and* T*) of the trabecula.

The APP allowed for the measurement of the swelling along one axis at a time. In Figure 1(e) the distilled water filled container for the measurement of the swelling of the trabecular length is shown. After the execution of each trial relative to the hygroexpansion along one main axis of the trabecula (i.e., length, width, or thickness) the specimens were dried up at room temperature and humidity for 72 hours; then the trial was repeated for the other trabecular axes. Each trial was repeated five times and the mean hygroexpansion over time was computed. Standard deviation of the measured swelling dynamics was less than 5%.

The experiments were conducted at room temperature and relative humidity of 41 ± 3%RH and 27 ± 1°C, respectively (mean value ± standard deviation).

The two outputs signals from both the load cell and the micropositioning controller were also connected into the data acquisition system. It was built using a PXI 1031 mainframe equipped with a PXI 5122,14-bit digitizer, and PXI 8176 embedded controller (National Instruments, Texas, USA).

The data analysis software was programmed* ad hoc* and recorded in NI Labview. The graphical user interface (GUI) provides the user with complete control over all aspects of the swelling versus time. In this way, the APP operates as a high sensitivity dilatometer.

### 2.3. Statistical Analysis

All values are expressed as mean ± standard deviation. The exact Wilcoxon test was used to evaluate the differences of elongation between the axial directions of the specimens. The exact Friedman test (more than two time samples) and the exact paired Wilcoxon test (2 time samples) were applied to test for changes between time points. Two-sided *P* values < 0.05 were considered statistically significant. A Bonferroni test was used to assess the changes in the elongation values for the two-tailed test. A value of *P* < 0.05 was significantly different. Statistical analysis [36] was performed using the Statistical Package for the Social Sciences for Windows (SPSS v. 20.0, Chicago, IL) program software package.

### 2.4. Theoretical Framework

The equations that describe the mass uptake over time in a porous sample of arbitrary shape immersed in a liquid [37] are analogous to that valid for the conduction of heat in solids [38]. The linear relationship between swelling and mass uptake is commonly accepted [39–47]. According to this approach, whose validity was confirmed by a comprehensive review conducted by [48], the experimental elongation data (Δ*L*_*m*_, Δ*W*_*m*_, Δ*T*_*m*_) measured along the principal axes *x*, *y*, and *z* of the specimen have been fitted using the exact solution for a conventional diffusion problem in a porous three-dimensional medium. The theoretical elongations (Δ*L*, Δ*W*, and Δ*T*) along *x*, *y*, and *z* at time *t*, obtained by the application of Fick's law [38], are given by

**Figure 2:**
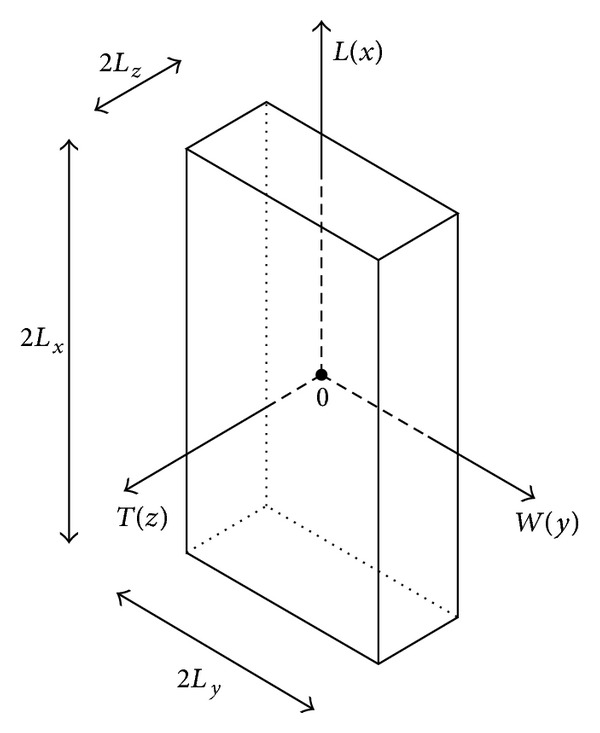
Sketch of the specimen geometry.

(1) ΔL(t)=∫−LxLxβx·{1−64π3∑n=0∞∑k=0∞∑p=0∞(−1)n2n+1·(−1)k2k+1·(−1)p2p+1·cos⁡[(2n+1)πx2Lx]              ·e−(Dx/4Lx2)[(2n+1)π]2·t·e−(Dy/4Ly2)[(2k+1)π]2·t·e−(Dz/4Lz2)[(2p+1)π]2·t}·dx,ΔW(t)=∫−LyLyβy·{1−64π3∑n=0∞∑k=0∞∑p=0∞(−1)n2n+1·(−1)k2k+1·(−1)p2p+1·cos⁡[(2k+1)πy2Ly]  ·e−(Dx/4Lx2)[(2n+1)π]2·t·e−(Dy/4Ly2)[(2k+1)π]2·t·e−(Dz/4Lz2)[(2p+1)π]2·t}·dy,ΔT(t)=∫−LzLzβz·{1−64π3∑n=0∞∑k=0∞∑p=0∞(−1)n2n+1·(−1)k2k+1·(−1)p2p+1·cos⁡[(2p+1)πz2Lz]      ·e−(Dx/4Lx2)[(2n+1)π]2·t·e−(Dy/4Ly2)[(2k+1)π]2·t·e−(Dz/4Lz2)[(2p+1)π]2·t}·dz,

in which *D*_*x*_, *D*_*y*_, and *D*_*z*_ and *β*_*x*_, *β*_*y*_, and *β*_*z*_ are the mass diffusivities and the linear expansion coefficients along axes *x*, *y*, and *z*, respectively, whereas 2*L*_*x*_, 2*L*_*y*_, and 2*L*_*z*_ are the dimensions of the specimen, as shown in Figure 2. In this last figure the reference Cartesian coordinate system (*x*, *y*, and *z*), whose origin is located in the center of the sample, is also represented.

The determination of the values of the unknown variables *D*_*x*_, *D*_*y*_, and *D*_*z*_ and *β*_*x*_, *β*_*y*_, and *β*_*z*_, which represents the main aim of the present experimental work, is carried out by means of a genetic algorithm. As well known, genetic algorithms belong to the family of optimization methods usually called evolutionary algorithms, which can handle nonlinear problems defined on discrete search spaces in a faster way compared with other optimization methods. To this aim, a specifically developed computer code based on a genetic algorithm is used to fit a set of experimental measurements of the swelling along the three principal axes of a trabecula with 2*L*_*x*_ = 9 mm, 2*L*_*y*_ = 2 mm, and 2*L*_*z*_ = 0.5 mm (denoted as single trabecula A).

The genetic algorithm generates the values of the unknown variables (the three mass diffusivities and the three linear expansion coefficients) that minimize the root of the mean square percentage errors between numerical results and experimental data, which is assumed as object function Φ:

(2) Φ=(∑i=1N{[((ΔL(ti)−ΔLmi)/ΔLmi)×100]2N+[((ΔW(ti)−ΔWmi)/ΔWmi)×100]2N+[((ΔT(ti)−ΔTmi)/ΔTmi)×100]2N})1/2,

where the summation is extended to the *N* measurements executed during time.

It is worth pointing out that although the value of each linear expansion coefficient could be obtained as the ratio between the elongation measured at steady state and the corresponding initial length, that is, Δ*L*_*m*_/*L*, Δ*W*_*m*_/*W*, and Δ*T*_*m*_/*T*, such direct calculation could lead to underestimated values if the steady state regime is not fully attained at the end of the experiment. Thus, it has seemed more correct to use such experimental values of the linear expansion coefficients to initialize the genetic algorithm and verify* a posteriori* that the results obtained are compatible with the experimental data. Similarly, the initial first-approximation values assumed for the unknown mass diffusivities (*D*_*x*_, *D*_*y*_, and *D*_*z*_) are those obtained by interpolating the experimental elongation data during time using the unidimensional transient solution for the mass transfer in a porous medium [37]. For each variable, the range of existence has been set by assigning a ±40% interval around its initial value. The simulation procedure ends when the relative difference of the object function Φ given by (2) between two consecutive generations of unknown variables is smaller than the preassigned value of 10^−2^. To ensure that the solution found does not correspond to a local minimum, several simulations have been run by increasing the range of existence of each unknown variable around its initial value up to a ±100% interval. No significant change has been observed in the results obtained. Further details about the genetic code used in the present work can be found in [49].

## 3. Results

For the plate-like trabecula A, the values of *D*_*x*_, *D*_*y*_, and *D*_*z*_ and *β*_*x*_, *β*_*y*_, and *β*_*z*_ obtained through the genetic algorithm are reported in Table 1.

**Figure 3:**
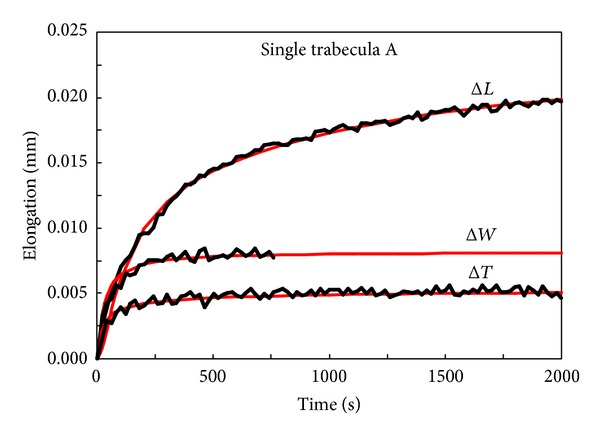
Comparisons between theoretical results (red line) and experimental data for single trabecula A.

The comparison between the theoretical elongations (in mm) versus time along *x*, *y*, and *z* of trabecula A, obtained from (1) using the values of *D*_*x*_, *D*_*y*_, and *D*_*z*_ and *β*_*x*_, *β*_*y*_, and *β*_*z*_, is listed in Table 1, and the corresponding experimental data is shown in Figure 3.

The validation of the results enumerated in Table 1 is carried out by reproducing numerically the experiments performed on three different trabeculae, denoted as B, C, and D, whose sizes along *x*, *y*, and *z* are specified in Table 2.

**Figure 4:**
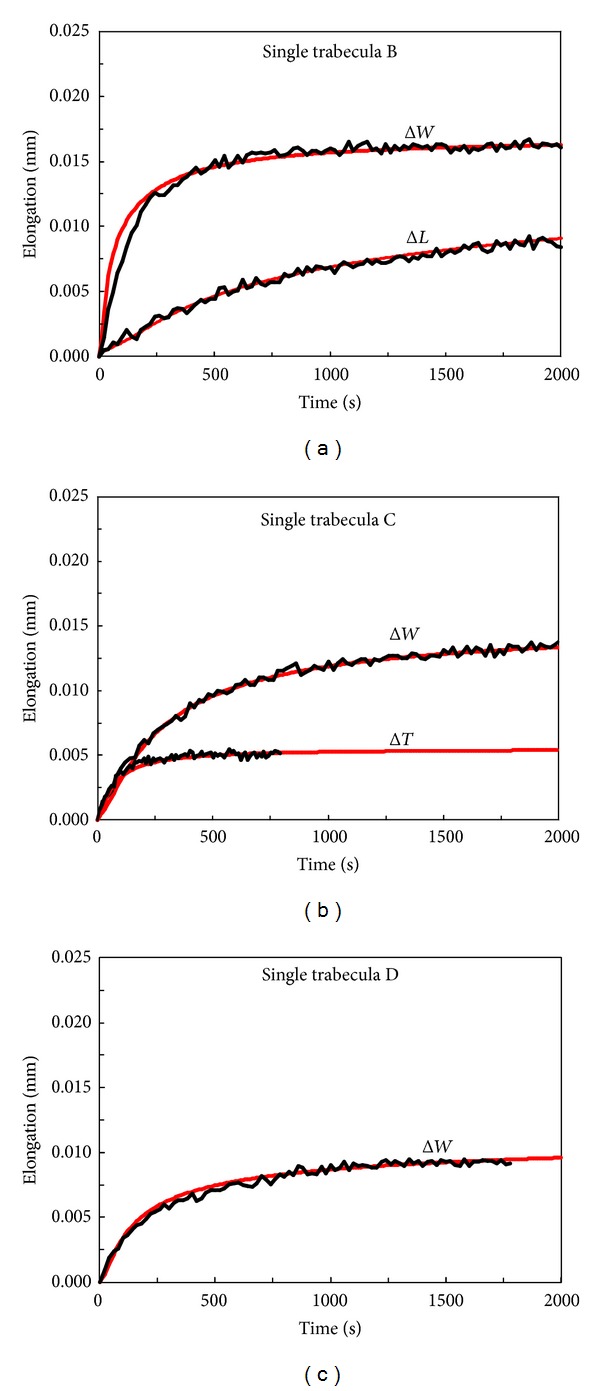
Comparisons between theoretical results (red line) and experimental data for single trabeculae (a) B, (b) C, and (c) D.

The distributions of the theoretical elongations versus time, obtained using (1), and those of the corresponding experimental data are reported in Figure 4. An excellent agreement was found, that is, less than ±5% of relative error.

## 4. Discussion

The fit performed via genetic algorithm and (1) is excellent for the plate-like specimen A, as depicted in Figure 3.

By now, the average value ± standard deviation for the apparent diffusion coefficient 3.56 · 10^−11^ ± 0.78 · 10^−11^ (m^2^ s^−1^) was measured using NMR for four cortical bone specimens from rabbit tibia by [27]. The analysis of the discrepancies with respect to similar studies is beyond the scope of this work and should be investigated considering the different arrangement of the lamellae in trabecular or in osteonal cortical bone [50–52]. Also different initial and final water content are likely to affect the measured swelling by [22]. However a simple description could be proposed to find some match of our results with those described in [27]. Cortical bone is organized as a twisted and rotated plywood structure [50, 52] whereas in the trabecular bone tissue the collagen is aligned along the trabecular main axis [53]. The mass flux* F* along the direction *r* according to Fick's law is expressed as a function of the concentration gradient *dC*/*dr* and the diffusion coefficient *D*_*r*_:

(3) $$\begin{array}{c} F_{r}=-D_{r}\frac{dC}{dr}. \end{array}$$

If along an axis the mass transport is equally subject to three different diffusion rates *D*_1_, *D*_2_, and *D*_3_, such as what could happen through a twisted and rotated plywood structure, the overall apparent diffusion coefficient *D*_*o*_ can be computed as follows:

(4) $$\begin{array}{c} D_{o}^{-1}=D_{1}^{-1}+D_{2}^{-1}+D_{3}^{-1}. \end{array}$$

In our case, substituting *D*_1_, *D*_2_, and *D*_3_ with *D*_*x*_, *D*_*y*_, and *D*_*z*_ of Table 1 yields *D*_*o*_ = 1.05 · 10^−11^, in fair agreement with that of [27].

An important finding was represented by the different behaviour exhibited by plate-like versus rod-like specimens. In particular, using *D*_*x*_, *D*_*y*_, and *D*_*z*_ found for specimen A does not allow a correct prediction of the swelling dynamics for rod-like trabeculae such as specimens B and D (Table 2) along some axis. This circumstance could be explained with a dissimilar nanostructure of the collagen-apatite porosity within the cross section, with respect to that of plate-like trabeculae. This in turn could be due to a marked anisotropy of the alignment of the apatite crystals caused by local loading conditions that, after remodelling, yielded a different momentum of inertia and thus flexural stiffness with respect to the plate-like trabeculae.

Regarding the measured linear expansion coefficients, the minor dimensional change along the axial direction is evident, which corresponds to the axis of the mineralized collagen fibrils, while the major swelling comes out along the thickness and width directions. This fairly agrees with the results of similar studies [54, 55].

## 5. Conclusion

We have illustrated the measurement of the swelling of single trabeculae from human femur heads during water imbibition. Moreover, since the swelling is caused by water diffusing from external surfaces to the core of the sample, by measuring the sample swelling versus time, we have obtained direct information about the transport of fluids through the intimate constituents of bone.

Our technique, based on the measurement of swelling, appeared actually sensitive on the water diffusion through the collagen bone matrix. As a consequence it may be argued that, in pathological tissue, deviations from normal fine structure reflecting in abnormal arrangements of collagen fibrils could be detected.

An important issue dealing with the present method is the quite cumbersome measurement of the specimen size since single trabeculae are often irregularly shaped yielding relatively dispersed results. However, these preliminary results are encouraging and suggest that further analysis on a consistent number of specimens could give a fundamental insight into the microstructure of bone tissue, depending on anatomical sites as well as normal or pathologic conditions.

To our knowledge, our study is the first measurement of the three apparent diffusion coefficient along each of the principal axis of human single trabeculae, revealing a marked anisotropy of transport phenomena within the Collagen Apatite bone matrix. In particular, the great difference among the measured diffusion coefficients *D*_*x*_, *D*_*y*_, and *D*_*z*_ could provide additional in-depth data on fibre arrangement in the lamellar bone system in order to give further evidence of the relationship between the orientations of the fibre bundles.

In conclusion, since metabolic activities, adhesion, migration, and thus a proper growth of cells require proper nutrient diffusion that is in turn affected by cell-matrix interactions [1], we believe that the present work can be a valuable support to the design of ECM-like scaffolds for bone tissue engineering to optimize the transport phenomena and the mechanical properties of bone substitutes.

## Figures and Tables

**Table 1:** Apparent diffusion coefficient and linear expansion coefficients for specimen A.

Directions	Apparent diffusion coefficients (m^2^·s^−1^)	Linear expansion coefficients
Length (2*L*_*x*_)	*D*_*x*_ = 1.03 · 10^−9^	*β*_*x*_ = 0.00195
Width (2*L*_*y*_)	*D*_*y*_ = 1.26 · 10^−10^	*β*_*y*_ = 0.0053
Thickness (2*L*_*z*_)	*D*_*z*_ = 1.16 · 10^−11^	*β*_*z*_ = 0.0107

**Table 2:** Single trabeculae sizes.

Single trabecula	Length—2*L*_*x*_ (mm)	Width—2*L*_*y*_ (mm)	Thickness—2*L*_*z*_ (mm)
B	7.9	2.6	2.5
C	6.3	2.3	0.5
D	7.2	2.1	1.7
